# Fiber chemistry and technology: their contributions to shaping Society 5.0

**DOI:** 10.1186/s11671-023-03888-4

**Published:** 2023-09-16

**Authors:** Mariam Al Ali Al Maadeed, Deepalekshmi Ponnamma

**Affiliations:** 1 Office of Vice President for Research and Graduate Studies, Qatar University, 2713 Doha, Qatar; 2 Center for Advanced Materials, Qatar University, 2713 Doha, Qatar; 3 Materials Science and Technology Program, Department of Mathematics, Statistics and Physics, College of Arts and Sciences, Qatar University, 2713 Doha, Qatar

**Keywords:** Society 5.0, Spinning, Technology, Industry, Smart

## Abstract

Society 5.0 establishes innovations and innovativeness as the basic platforms for accelerating the development of solution-based strategies for the sustainability problems every society is facing. It features an interactive cycle operating at a society-wide level through which data are collected, analyzed and transformed into applicable technology for the real world. Transforming the current society into a super smart society requires in-depth knowledge of the Internet of Things, robotics and artificial intelligence. Being a member of the 4th industrial revolution is significant; however, it is equally important to alleviate the socioeconomic challenges associated with it and to maintain sustainability. From cellulose to carbon, fibers have utmost importance in technological applications, industrial developments and sustainability. Fibers are identified as useful energy resources, water treatment mediums, supercapacitors in electronic devices and wearable e-textiles. Therefore, knowing the chemistry behind fiber manipulation for advanced applications for Society 5.0 is beneficial. In this paper, we highlight the contributions of fibers to shaping Society 5.0 and their modifications and role in providing a sustainable environment. We highlight the chemical aspects behind tailoring fibers to provide state-of-the-art information on fiber-based products. We also provide background information on fiber technology and the sustainable development goals for a fiber-oriented Society 5.0. Scientists, researchers and specialists in this field should understand the impact of tailoring and influencing society as a whole.

## Main

Society 5.0 emerged as a future human-centered super smart society in the 5th Science and Technology basic plan that Japan aspires to adhere to [1]. Society 5.0 addresses all challenges faced by Society 4.0, such as inadequate cross-sectional sharing of knowledge, difficulty in collecting and analyzing information, age-restricted labor and action, aging populations, and cooperative difficulties [2]. Personalized actions are expected to be used, thus optimizing the complete social and organizational system. Society 5.0 can offer comfort, vitality and high quality standards through the digital transformation of existing societies. However, prior planning in regard to the following is needed: (1) national strategies, (2) legal regulations to push administrative digitization, (3) identifying all technologies for economic growth, (4) broadening human resources with advanced digital skills, and (5) social implications, ethics and social acceptance by all stakeholders.

Society 5.0 can be realized only by strategically utilizing innovations such as IoT, big data, robotics, AI and the circular economy. These were the major components in the fourth industrial revolution (Industry 4.0). The big data collected through the IoT are used to establish new levels of intelligence to reach people in every nook and cranny of society in the correct amounts at the required time. In fact, Society 5.0 and Industry 4.0 are complimentary, as the former uses technologies and intelligent systems from the latter and enhances the benefits [3].

The potentialities of Industry 4.0 include personalized consumer interactions, focused technological solutions for each sector, specific product manufacturing at low volumes, constant value chain changes by high competitiveness and flexibility, high operation efficiency and productivity, low energy cost and work-life balance. It connects people, objects and equipment systems through an intelligent real-time network of digitalization and data exchange. Smart objects used in industry enhance resource efficiency and adaptability and integrate data transmission and production processes. Resource usage in an efficient and controlled way based on an intelligent, cross-linked, value-creation framework is significant for maintaining sustainability. Regarding sustainable value creation, three main sectors are considered, the environment, society and economy, as structural areas for essential and integrating arrangements. This framework supports global supportability goals and applies reasonable measures to promote trade models, creation systems and modules.

The objectives of Society 5.0 are concentrated on sustainability development goals (SDGs) [4]. The sustainable competitiveness of an enterprise is viewed as socioeconomic sustainability in the short and long term, specifically in terms of competitive advantages, such as influence, innovative potential and efficiency, availability and use of labor, building innovation capability, etc. [5]. Sustainability is regarded as achieving high production in a short time based on the above fundamentals and social, ecological and economic balances. Society 5.0 seeks to achieve smart, innovative and quick economic growth in line with the sustainable development targeted by United Nations Organizations up to 2030. Therefore, in addition to safety, energy efficiency and ecofriendliness as the primary goals, physiological and hygienic environments, stable environments and industrial independence for global marketing are also goals of sustainable competitiveness. The transition to Society 5.0 will require strengthening human resources and education, infrastructure, information security, research facilities, smart manufacturing and regulatory measures.

Our aim in this perspective is to show the role of fiber technology in supporting the development of a sustainable and smart Society 5.0. We begin with a general description of Society 5.0 and its relation with Industry 4.0 and the SDGs. Then, we explain the art of fiber formation and the chemistry behind it. After that, we show examples of the ways in which fiber has contributed to society. The paper is concluded with the way forward and a summary of fiber technology contributions to SDGs. Advances in fiber technology and the chemistry behind fiber modifications are reviewed elsewhere [6–8]. However, the applications of fiber reinforcement and modifications for building the interconnection between Industry 4.0 and Society 5.0 are discussed in this study. A critical analysis of various factors influencing fiber properties and the challenges in achieving sustainable goals are mentioned.

## Background

Society 5.0 follows from the previous versions of society: the hunting society 1.0, agrarian society 2.0, industrial society 3.0, and information society 4.0. This society addresses all social problems in the most feasible economic way by integrating physical space and cyber space [9]. This society has its origin in Japan's effort to reduce their current challenges in industry and technology. Due to the unique advantages of the abundant accumulation of operating data from numerous manufacturing facilities and healthcare systems and advanced technologies using AI and big data, Japan aims to overcome challenges such as community aging, a decrease in the productive age population and energy and environmental challenges [10]. For example, robotics and remote healthcare facilities are essential in society. Other important areas include medical data, autonomous public transportation, drone-controlled vehicles and inspection and maintenance using sensors, AI and robots. All of the above practices can increase safety and productivity while reducing costs and required efforts.

Society 5.0 enables organizations to adapt and prepare for change and qualifies stakeholders for their active role in sustainable development and in creating a new technological environment. Technical solutions that substitute natural resources to reduce greenhouse gas emissions, the loss of food, the revolutionary rate of production and sustainable industrialization are encouraged in this smart society. This socially responsible development among stakeholders is beneficial for social well-being and advances sustainable economic systems [11]. Unlike Industry 4.0, Society 5.0 includes advanced analytics (with smart machines) and people as elements and goes beyond production and thus can boost economic activity. While machines work with less complex software in every part of production, advanced analytics provide extensive knowledge by integrating physically advanced algorithms and automation. Robotics is a major area in Society 5.0, as it requires zero downtime with maximum efficiency. It provides a smart working environment by sensor-assisted automation. The major milestone to achieve in Society 5.0 is automation, which is based on virtual artificial structures consisting of artificial systems (A), computational experiments (C), and parallel execution (P) [12]. In Society 5.0, the management components should broaden product outreach without the need for conventional marketing [13].

Industry 4.0 offers real-time production planning and dynamic optimization by revolutionizing products and services through increased operational efficiency and developed business models [3]. It is described by three paradigms: (1) collective production strategies and resources for achieving intelligent products with individual operations data and standard memory; (2) decentralized self-organization to achieve flexible, modular and productive intelligent machines; and (3) skilled and augmented operators for automation. The main components in Industry 4.0 are cyber physical systems, the Internet of Things and the Internet of Services [14]. The major resources, as in any other industry, are materials, energy and water. One of the major challenges in Industry 4.0 is the cost-quality balance. Productive, flexible and efficient methods are employed to guarantee quick responses to social problems by innovative means. For instance, Industry 4.0 incorporates additive manufacturing as a major paradigm, as it can be used to develop new technological inventions by replacing old manufacturing methods [15, 16].

Both Industry 4.0 and Society 5.0 emphasize the IoT, AI and big data analysis and facilitate a top-down, state-led approach by collaborating with the academic, industrial and government sectors [17]. However, Industry 4.0 focuses on smart manufacturing and factories, while Society 5.0 aims for a super smart society as a whole. The key phrases used in Industry 4.0 include cyber physical systems, mass customization, etc. In Society 5.0, the key phrases include high-level physical-cyber space convergence, balancing economic development with the resolution of social issues and a human-centered society. One of the main challenges to overcome in Society 5.0 is to optimally balance people's needs with those of society. The regulations and technical bottlenecks in constructing cyber architecture and achieving international standards and security measures are also considered as some of the challenges to resolve in Society 5.0. The main ideas of Industry 4.0 and Society 5.0 can be summarized as digital manufacturing and a digital society [18].

Sustainable development goals (SDGs) were developed by the United Nations as an urgent call for action to protect the people and the globe, now and in the future, through global partnerships [19]. The list of SDGs was made to strengthen health and education, end poverty, reduce inequality, address climatic change, preserve forests and water, and spur economic growth. These aspects are classified into 17 goals, all leading to the sustainable development of human society. While the SDGs that are achievable through fiber technology are (1) SDG6: Clean water and sanitation; (2) SDG7: Ensure access to affordable, reliable, sustainable and modern energy for all; (3) SDG9: Increase industry, innovation and infrastructure; (4) SDG11: Ensure sustainable consumption and production patterns; and (5) SDG 13: Take urgent action to combat climate change, their impacts directly influence Industry 4.0, Society 5.0 is influenced by all of them (all 17 SDGs).

**Fig. 1:**
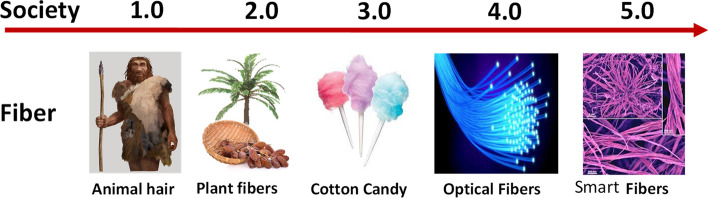
Schematic representation of different fibers used in various societies

Fibers have a long history of being included in technological applications, including in the aeronautical, medical, military, electronic and textile fields. They are one of the primary materials humans use for all of their basic needs: shelter, food and clothing. Figure 1 schematically shows the growth of fiber technology with various societies. While animal fibers were used as clothing material for hunters, fibers of various plant origins were widely used by agrarians. The industrial revolution also included manufacturing fibers, such as cotton candy, which later paved the way for optical fibers and very recently nanofibers.

## The art of fiber formation and the chemistry behind it

The evolution of fiber technology began in the Stone Age, in which human beings hunted animals for their meat and skin. While the meat satisfied their hunger, animal skin was used to protect themselves from severe climatic conditions. This usage of animal hair as clothing continued throughout the primitive hunting society and even continues today in an evolved form. With the passing of time, agriculture emerged as the major source of food, income and living, and humans started converging in to localities with living less like nomads. This settling for the sake of agriculture was the basis of forming societies and required comfortable shelters to live in. Plant fibers satisfied most of these demands, as leaves were used to form the roofs of shelters, wood was used to form stronger construction materials, and fruits and seeds were used as the food. Fibers developed over the years, their mechanism of formation improved, and their application levels increased throughout societies. During the past few centuries, new chemistries and techniques have been adopted for fiber formation, with nanofibers as the current frontier technology, having been developed from macro- and microfibers.

Silk was considered an expensive fiber in the seventeenth and eighteenth centuries and was used in the high-fashion textile industry [20]. The discovery of artificial silk, and glass fibers at the beginning of the nineteenth century was historically significant in terms of the history of fiber, through which the art of fiber formation by drawing through an orifice was identified. In the first half of the nineteenth century, the world witnessed several efforts to manufacture fine fibers for various applications, for instance, the Chardonnet process for cellulose fibers, viscose process for fiber production from alkali-cellulose, hot stretch process for rayon manufacturing, etc. [21]. The discovery of macromolecules or polymers was in fact greatly revolutionary in fiber history, specifically when nylon fibers were made by DuPont in 1926 [22]. Following this, many synthetic polymeric fibers were developed, such as polyesters, polyamides and perlon. Outstanding fibers such as Kevlar, carbon fibers, polyimide and aramid fibers were discovered during the late nineteenth century, and their superior mechanical, heat-resistant, and flexibility properties were revealed. Since 1950, with the discovery of glass fibers, optical fibers and other fibers, such as basalt, ceramic and carbon fibers, fiber-reinforced composites have been employed for engineering applications in various disciplines, such as in the fields of materials, biomedical, textiles, mechanical and chemistry.

**Fig. 2:**
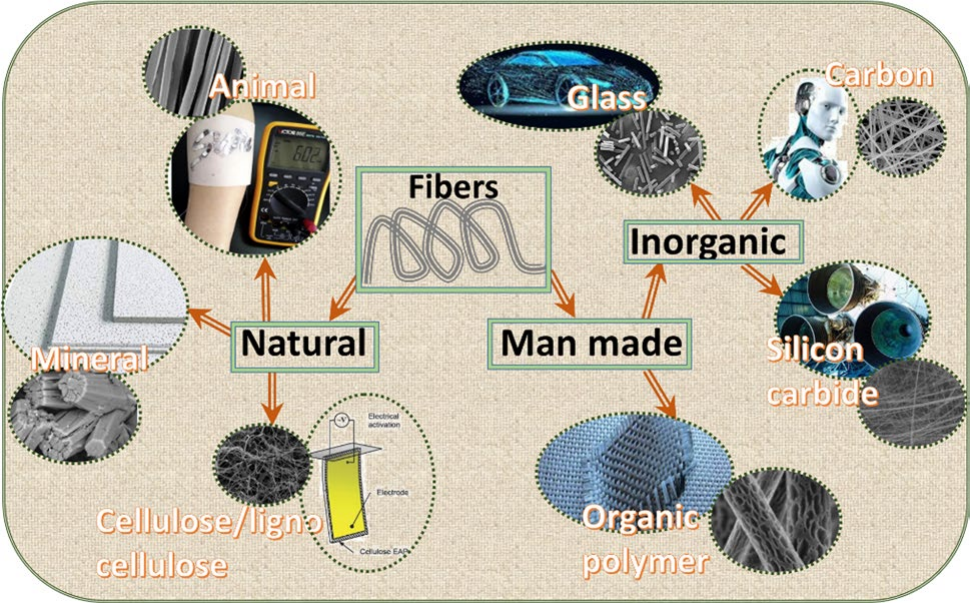
Classification of fibers and their applications in smart society

Figure 2 shows a schematic illustration of various kinds of fibers (according to their origin) and examples of their technological applications in Industry 4.0 (with regard to Society 5.0). Since Society 5.0 aims to develop advanced applications based on AI and IoT technologies, different kinds of polymeric, nanomaterials, and natural fibers can be applicable in sustainable and smart devices, especially in the fields of transportation, energy, health and defense.

### Types of fibers

*Natural fibers* Hair-like structures originating from animals, plants or mineral sources are natural fibers [23]. They are widely used in textiles and other industrial applications due to their water absorbing properties, flexible and comfortable designs, and high elasticity. While animal fibers (silk, wool, catgut, and alpaca) are composed of proteins, plant fibers (cotton, jute, flax, bamboo, date, coconut, sisal and hemp) contain cellulose. Other than their low environmental impact, the major advantages of natural fibers include renewability, light weight and flexibility, low cost, nonabrasiveness to processing equipment, better crash absorbance, and sound insulation properties. Although plant fibers have greater strength than animal fibers, aging, strain-rate sensitivity, weather-dependent chemical composition, low thermal stability, poor crease resistance, fiber shedding and yellowing, brittleness and high moisture content negatively influence the properties of both of these fibers.

*Chemistry* Plant fibers have a complicated layer-by-layer structure with a lumen, which is the main reason for their water absorption behavior. In fact, the middle layer or secondary wall is responsible for their outstanding mechanical properties and maintains their physical strength by firmly attaching to the entire micron-sized fiber [23]. Plant fibers contain different constituents, such as lignin, cellulose, hemicellulose, pectin, and wax. In its structure, the primary wall contains randomly oriented microfibrils, the secondary wall is composed of oriented, helical, winding structured microfibrils and the internal wall has a crystalline structure [24]. Cellulose microfibril walls are coated with hemicellulose constituents through hydrogen bonding. Cellulose consists of D-gluco-pyranose rings connected through β-(1–4)-glycosidic linkages, and its amount varies from fiber to fiber. Flax, jute, cotton, and hemp contain 70–96% cellulose, whereas coir, bamboo and bagasse have 20–45% cellulose in their structure. Hemicelluloses, a group of complex polysaccharides (glucose, mannose, arabinose and xylose), form covalent bonds with cellulose and H-bonds with lignin. These amorphous groups cause low thermal stability and high absorption capacity in the plant fibers and have an abundance of –OH groups in their structure. Lignin consists of aromatic and aliphatic hydrocarbon groups and enhances the stiffness, chemical adhesive nature and resistance to microbiological attack of plant fibers. Pectin (heteropolysaccharides) endows plant fibers with wall porosity and flexibility, and lipids, wax and fats prevent plant fibers from drying. In addition, waxes and long-chain ester alcohols negatively affect the wettability and quality of plant fibers.

*Animal fibers* Fibers of an animal origin, such as feathers, wool, silk, hair, etc., are less readily available and thus have a high cost compared to plant fibers. Since animal fibers are protein rich, they can act as protectors for cells and tissues and are elastic and capable of scaffolding and stabilization. For this reason, they are mostly used for biomedical applications, except wool and silk, which are mostly used in textile industries. α-Keratin is a complex structured component of animal fiber with a nonuniform chemical composition. Compared to plant fibers, animal fibers possess a greater water absorbing capacity and high resistance to alkali medium.

**Fig. 3:**
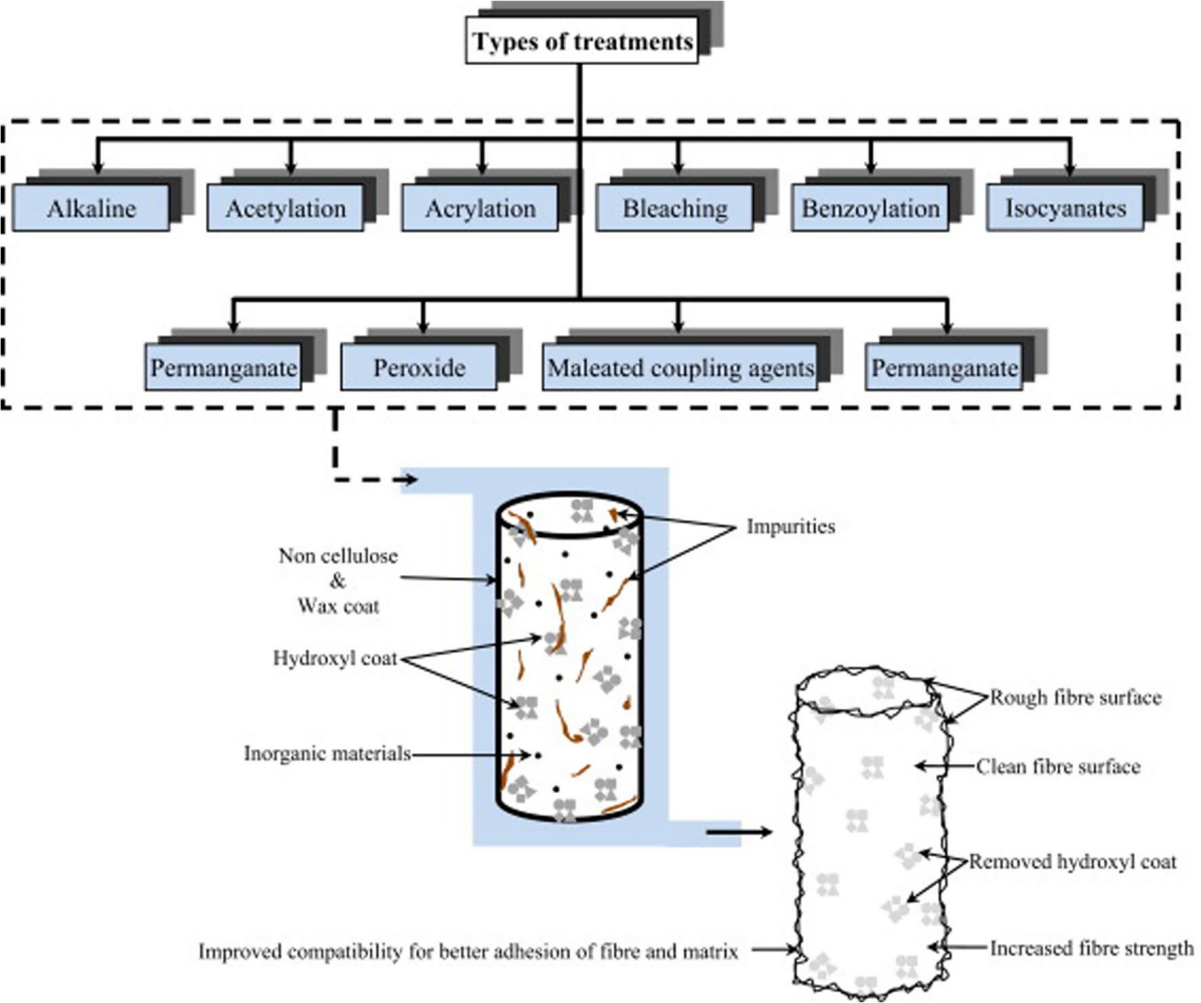
Different types of treatments of natural fibers [23]. Copyright 2015. Reprinted with permission from Elsevier

*Chemistry* Natural fibers (both plant and animal origin) are extracted through manual extraction, retting methods and mechanical extraction processes, which often take days to months to complete [24]. However, the chemical retting process using alkali treatment is rather easy and produces fibers of good quality. Various chemical treatments are applied to extract the fibers as well as to induce their surface modification, as schematically represented in Fig. 3 [23]. In all reactions, the main purpose is to remove the -OH groups from the fiber surface and thus to increase the surface roughness.

$$- {\text{OH}} + {\text{NaOH}}{-} > {\text{Na}}^{ + } {\text{O}}^{ - } + {\text{H}}_{2} {\text{O}}$$

In addition to the –OH coating, the noncellulosic components in plant fibers, inorganic materials and wax can be removed by surface treatments. Alkaline treatment cleans the fiber surface, results in lower surface strains, removes most of the amorphous fiber parts and improves the adhesion properties of fibers. In acetylation [25], acetyl –CH_3_CO groups are used to remove –OH groups and reduce hydrophilicity, in addition to decreasing the dampness retention and improving the life of the fiber. While benzoylation incorporates benzoyl (C_6_H_5_C=O) groups on the fiber to improve epoxy adhesion and surface roughness, maleated coupling agents (maleic anhydride) catalyze esterification and H-bond reactions at the fiber surfaces. Acrylation and acrylonitrile grafting enhance the coupling performance of fibers in a specific matrix, and permanganate and triazine treatment enhances adhesion. Hydrolyzed silane solution treatment with natural fibers causes the formation of Si–O–Si bonds among silanol groups and H-bonds with natural fibers [24]. Silane treatment changes the fiber morphology/dimension, makes the fiber porous and reduces water absorption. Bleaching and stearic acid treatment can remove the noncrystalline constituents from the fiber structure and result in better fibrillation by separating the fiber groups. Among all fiber treatment methods, alkali treatment is simple and effective in generating high interfacial adhesion properties.

*Mineral fibers* Fibers such as asbestos, wollastonite, and fibrous brucite are accessible naturally or in customized style. While asbestos is a natural fiber, rock wool and slag wool are synthetic mineral fibers. Mineral fibers of asbestos are notable for their high tensile strength, poor heat conduction, high electrical, alkali, and acid attack resistance and sound absorption capabilities [26].

*Chemistry* Chemical treatment of asbestos includes highly basic pH to produce silanols and hydrofluoric acid treatment to produce silicon fluoride. An operating temperature of 100 °C, high cost and wastewater disposal are considered some of the disadvantages of this method. Moreover, asbestos has a great environmental impact, making it a material of low choice for building Society 5.0.

**Fig. 4:**
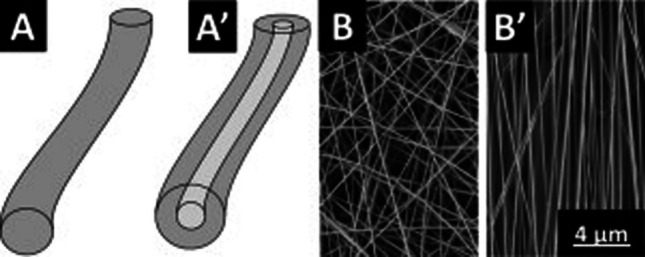
Scheme of **A** uniform and **A′** core–shell nanofibers and SEM images of **B** randomly oriented and **B′** aligned nanofibers [27]. Copyright 2015. Reprinted with permission from Elsevier

*Fiber-forming polymers* Linear and branched polymers are formed through strong covalent bonds between the monomers. The molecules also possess intermolecular forces or van der Waals forces, due to which they have good solubility in specific solvents and soften when melted. Linear polymers and a few branched polymers form fibers in a solution or molten state. However, highly complex network polymers that undergo chemical decomposition without melting and swollen structures and without dissolving in solvents are not suitable for fiber formation. Polyester, polypropylene, polyethylene, polyurethane, polyamides, polyacrylonitrile, aramid and cellulosic polymers are examples of good fiber-forming polymers. However, sometimes not all desired properties are obtained from polymeric fibers; for instance, the tensile strength of polypropylene fibers is only approximately one-fourth of that of high-modulus polyethylene fibers. Certain additives, such as heat and light stabilizers, flame retardants, TiO_2_, dyes and pigments, often overcome these lackluster properties. Polymeric fibers also form in nanodimensions to generate nanofibers with outstanding mechanical and flexibility properties [27]. Depending on the structure, nanofibers can be uniform solid, core–shell and hollow fibers and based on orientation, aligned and randomly oriented (Fig. 4). Depending on the desirable properties and applications, materials are selected and processed for fiber generation. For instance, chitosan-based nanofibers are resilient, mechanically strong, mucoadhesive and antimicrobial and are applied in tissue engineering.

*Chemistry* The prior condition for fiber formation by a polymer is being in a liquid or semiliquid state, either by melting or by dissolution. Long molecular chains are released from the van der Waals forces, and the independent chains are extruded through a small orifice in a spinneret. Fine jets of liquids solidify into long fibers or filaments, and the overall process is called spinning. Solution spinning includes wet and dry methods, both involving drying the viscous polymer solution jets. However, wet spinning involves a spin bath into which the solvent from the extruded material diffuses out; at the same time, a nonsolvent diffuses into the extrudate. The polymer coagulates and thereafter stretches into rolls and packages. Dry spinning moves the polymer solution into a heated column, wherein direct solvent evaporation and fiber solidification occur. In the most economical method of melt spinning, viscous polymer melt is extruded through a spinneret of many holes, and cold air is used to solidify the fiber. There are also other methods, such as gel spinning (highly viscous polymer solutions are spun in a near-semisolid state) and emulsion spinning (nonmelting and insoluble polymers are ground and suspended in another polymer solution for spinning). Split film fibers are also made by cutting ribbons of polymers into fine fibers.

Drawing is a simple fiber generating method in which a fiber is drawn and solidified from a sharp tip with a polymer solution droplet. It is discontinuous, and the product amount is also limited. There are also other methods to create fibers of microdimensions, such as force spinning using centrifugal force, melt blowing through an extrusion process, interfacial polymerization through monomer-anion aggregate formation at an immiscible liquid‒liquid interface, and phase separation by a solvent exchange process for a polymer gel [27]. Melt blowing also produces microdiameter fibers by generating a shear flow mechanism (sliding movement of layers) [28]. Fiber attenuation involves different monolayer arrangements with longer lengths before the fiber draws itself and lowers the fiber tenacity. A combination of techniques, such as the sandwich structure of nonwoven materials, a spun bond fiber-web on two sides, and a melt blow fiber-web in the middle, can enhance the tenacity of fiber-web products. Electrospinning also produces nano- and microfibers of other materials by using typical polymers as templates. For instance, silicon carbide microfibers were made from polycarbosilane and later pyrolyzed to separate the semiconducting fibers. This occurred when CH_4_, H_2_ and CO gases evolved during the chemical reaction between the –(CH_3_)S– bonds among molecules with heat treatment. With temperature, the organic polymer structure completely converted into a Si–C– bonded structure. The pipe-shaped fibers had minimal fracture and pores and were applied in electronic devices for high-power switching and in high-voltage light emitting diodes [29].

*Optical microfibers* Optical fibers are one-dimensional hair-like glass filaments of any suitable polymer [30]. They can function as an efficient energy and image transmission medium for computer networks. Although the scope of optical fibers is already beyond defense, imaging, biomedical technology, etc., their surface modification and functionalization can enhance their fundamental properties to further broaden their applications to sensors, electronics and robotics. The core and cladding with different refractive indices, which facilitate the propagation of incident light, are important components of optical fibers. Polymer optical fibers are another class of optical fibers modified using polymers (e.g., polystyrene, polymethylmethacrylate, polycarbonates, cyclic olefin copolymers and amorphous fluoro-polymers). Transmittance, refractive index, photosensitivity, and transmission losses are significant properties of optical fibers influenced by polymer modification.

*Chemistry* Chemical modification of optical fibers includes dip coating, electrospinning, vapor deposition, crucible melting and plasma processing [30]. In dip coating, complex U-shaped substrates can be immersed in a specific chemical agent, the metal ions of which deposit on the substrate surface. For example, dipping in metal alkoxide solution develops a metal oxide layer, and in iron (III) nitrate/ethylene glycol/2-methoxy ethanol, nickel ferrite is developed. Electrospinning orients the functional groups of chemical agents on the fiber surface and makes their extraction comparatively easier than that under dip coating. High optical absorption, elastomer infiltration and ultrasound generation allow optical fibers to be applied in pulse echo imaging. Chemical vapor deposition to modify the surface with rare earth elements, a crucible melting process to strengthen the silica cladding by lanthanum/aluminum codopants and plasma processing to produce highly ionized species on the fiber surface are also practiced. Such optical fibers have high application potential in sensing, imaging, photodynamic therapy, information technology, laser surgery and telecommunication.

*Fibers of nanomaterials* Nanofibers are fibers on the nanoscale with a very high aspect ratio, controllable pore structure and good pore interconnectivity [31]. Carbon fibers, as the name indicates, are made of a carbon backbone, and their properties vary according to the nature of the precursors used for synthesis [32]. They are classified as PAN-based, mesophase and isotopic pitch-based, and rayon-based. Carbon fibers, including vapor-grown fibers, are mechanically strong, lightweight and resistant to corrosion. Carbon fibers are already used in several applications, such as sports, building materials, automobiles, aircrafts, and power generation systems. Carbon fibers possess inherent anisotropy due to the preferential orientation of graphene planes along the axis. While van der Waals forces exist between the graphene sheets, electrochemically different edge-oriented graphite is observed at the fiber edges. The conductivity of carbon fibers is 2–10 S/cm; however, single carbon particles pierce through the separators and cause electric short circuits.

*Chemistry* Nanofibers of unlimited length and diverse structural designs, such as core–shell, hollow and solid fibers, are developed through the electrospinning (high voltage to produce fibers from a viscoelastic polymer solution) method. Amphiphilic molecules can also self-associate through a self-assembly method to produce ultrathin nanofibers, but this method does not allow control over orientation and morphology. This is rectified by template synthesis, through which electrochemically or chemically assisted oxidative polymerization generates fibers on a nonporous support. Electrospinning results in various structural features of nanofibers depending on the significant experimental parameters, such as the applied voltage, needle diameter, needle tip to collector distance, nature and dimension of the collector used to deposit the fibers, etc. The specific parameters also depend on the fiber diameters; for instance, increases in polymer concentration, polymer molecular weight, viscosity, flow rate and nozzle inner diameter increase the nanofiber diameter. However, increases in the applied voltage, conductivity, dielectric constant and relative humidity cause the fiber diameter to decrease [27]. In addition to electrospinning, other methods are also used to produce nanofibers [31], such as ultrasonic deacetylation of cellulose acetate to form a fiber web, aqueous/organic interfacial polymerization to form polyaniline fibers, and single capillary electrospinning and calcination to form Ca^2+^/Au codoped SnO_2_ nanofibers.

Carbon fibers are synthesized from precursors of high crystallinity that have a decomposition ability without melting and are easily spun into filaments with minimum volatile carbon production during pyrolysis [32]. The chemical process involving the synthesis of carbon fibers involves the same procedures as stabilizing treatment, carbonizing heat treatment, and high-temperature graphitizing treatment. According to the orientation of graphite platelets along the fiber axis, carbon fibers can be classified as ribbon-like (parallel alignment), platelet-like (perpendicular orientation to fiber axis), and herringbone or fishbone-like (layers oriented at an angle). The high electrical and thermal conductivity of carbon fibers make them applicable in electronics and adsorbents and endows them with high mass transport behavior.

## Fiber surface modifications

Fiber surfaces are modified by various methods to tailor the applications of smart fibers. Surface modifications are done physically or chemically to improve the fiber-matrix adhesion and to induce specific surface properties such as hydrophilicity/hydrophobicity to the fibers. Improved fiber-matrix adhesion can be achieved in natural fiber composites by dewaxing, alkali treatment, isocyanate treatment, peroxide treatment, vinyl grafting, bleaching, acetylation, and treatment with coupling agents, as reported [33]. Such surface-modified fibers can produce polymer composites for automotive applications, biocomposites for environmental applications, etc. The general consensus is that covalent chemical bonding, acid–base interactions or hydrogen bonding, surface energies that favor complete wetting of the fibers, large specific surface areas of fibers, and surface roughness that permits lock and key type mechanical bonding are all effective ways to achieve good adhesion [34]. Proper surface modifications ensure the smart fibers' functional qualities and long-term performance. Photon-based processes are reported to modify fiber surfaces chemically and morphologically, depending on the radiation spectrum range and the radiation sources' properties [34]. Micro-roughening of fiber surfaces and photo-chemical surface modification employing monochromatic UV lamps enhance the mechanical properties of fiber-reinforced composites for their applications in smart textiles. Layer by layer method applies thin, multilayered coatings to fibers to make functional materials for regulated drug release, barrier qualities, or sensing capacities. For instance, a negatively charged polyelectrolyte and a positively charged drug-loaded polymer are alternately placed on the fiber surface to create a smart drug delivery fiber [35]. Due to the multilayered coating, the medicine can be released under regulated conditions in reaction to certain triggers, such as pH or temperature changes. An efficient and practical method for creating fiber-based, strain-sensible wearable sensors is a surface modification on non-conductive fibers, yarns, or textiles [36]. Surface depositions, printing, spray and rod coatings, dip coating, and roller coating can all be used to implement this strategy. Wide operating range and exceptional sensitivity are two benefits of the coating method for strain sensors. Due to the unstable coating layer created by strain stimulation, these devices' long-term performance stability is less than ideal; as a result, encapsulation is frequently required. The substrates of stretchy fabrics and inelastic fabrics enclosed in elastomers, are modified with conductive materials like metals, graphene, CNTs, and conductive polymers for strain sensors for advanced applications. Thin films or coatings are applied on fibers using the vapor-phase deposition technique known as chemical vapor deposition (CVD). It is frequently used to insert nanoparticles onto the surface of fibers to add functional coatings like hydrophobicity, anti-corrosion characteristics, or nanoparticles. A layer of a hydrophobic material, such as a fluorinated polymer, is coated onto the fiber surface via CVD to produce a smart superhydrophobic fiber [37]. With this coating, the fiber becomes self-cleaning and water-resistant, making it ideal for use in outdoor textiles or protective apparel. A controlled radical polymerization method called surface-initiated atom transfer radical polymerization (si-ATRP) is used to graft polymer chains from the surface of fibers. With the use of this technique, the length and structure of the polymer chains may be precisely controlled, resulting in fibers with specific mechanical, thermal, or chemical characteristics. A conductive polymer, such as polyaniline, is generated from the fiber's surface in a smart textile application utilizing si-ATRP. As a result, a conductive coating is produced, which can be utilized to sense various environmental elements like humidity or tension [38]. Although the fiber surface modifications create advanced applications such as health monitoring, pharmaceutical, and tissue engineering for smart society, their long-term stability and durability—especially under significant deformation—are always subpar due to a lack of protection, which requires further optimization.

## Fiber contributions to Society 5.0

**Fig. 5:**
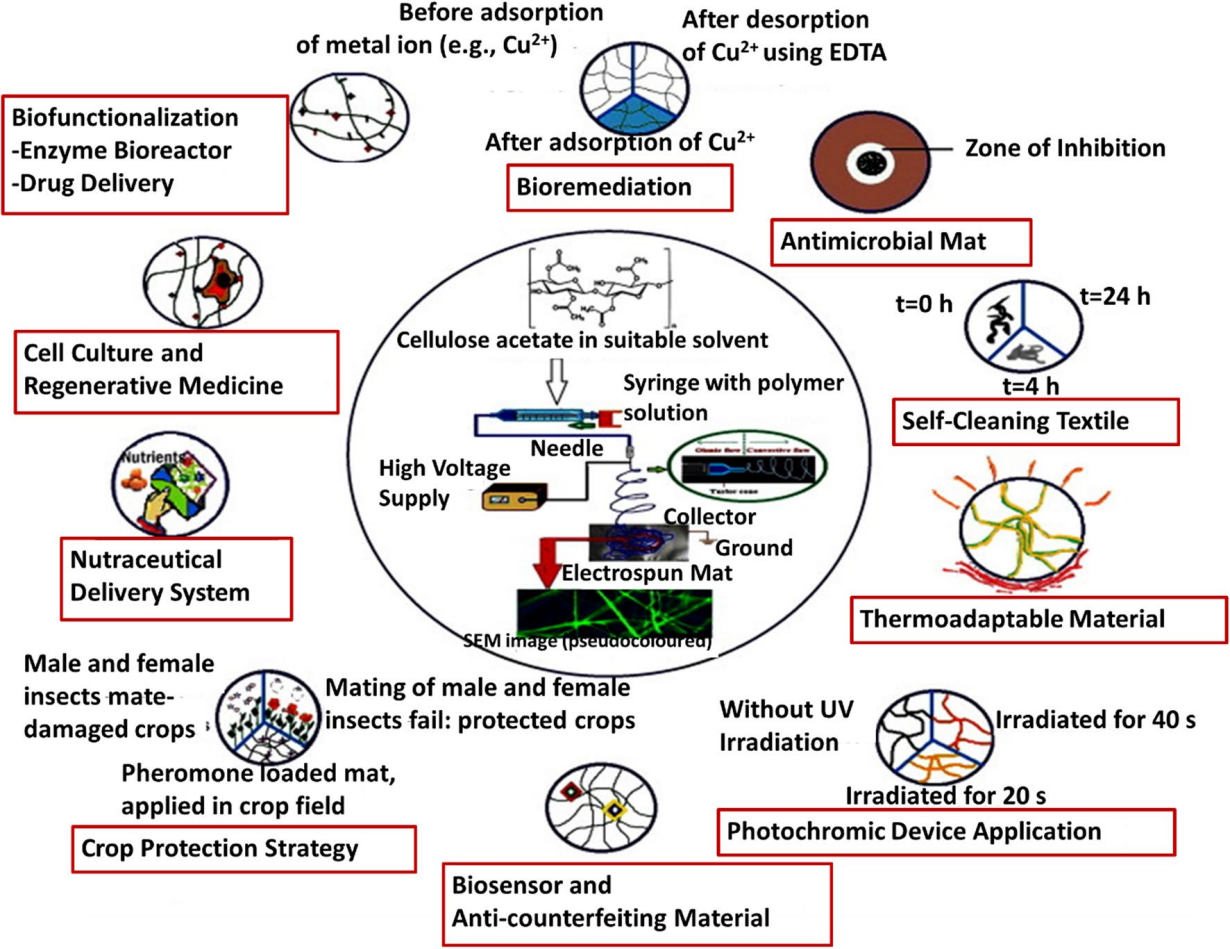
Applications of electrospun cellulose acetate fibers in smart technology [39]. Copyright 2013. Reprinted with permission from Elsevier

In Society 5.0, all of the abovementioned types of fibers will continue to play their roles in different applications and smart functions. They have special roles in designing different devices for high-profile applications. Electrospun fibers are useful in numerous areas; as an example, the applications of cellulose acetate are shown in Fig. 5 [39].

The development of fiber technology with the support of IoT connectivity and AI will reduce time-consuming data analysis. Robotics, AI, energy savings, sustainability, health and automation are considered important pillars in this social reformation strategy. Below, we discuss selected examples of the roles of fibers in supporting these pillars, including educational needs and contributions to SDGs.

- Sustainability

Ecofriendly materials and technology, using hybrid bioproducts from natural resources [40], realize sustainable development in Society 5.0. The sustainability of a product can be defined by many factors influencing its production and service, such as energy consumption, solid waste generation, depletion of natural resources, air pollution, global warming and land degradation processes. End of life, e.g., possible recycling, is an important parameter to be considered in every product design. In the automotive industry, lightweight and strong biomaterial-based structures are preferred to decrease fuel consumption and thus control CO_2_ emissions. Natural fibers from sisal, jute, hemp and kenaf are notable for their specific strength and modulus and low cost, and numerous composites are applied in automotive, aircraft and construction areas. Fibers of plant origin can be divided to the following groups: seed, leaf, bast, grass and core fibers. Kenaf fibers are well known for fabricating polymer matrix fiber composites, in which the fibers impart strength and stiffness by hydrogen bonding with themselves. From economic and environmental perspectives, kenaf is advantageous, as it can grow in almost all weather conditions. Other than the low cost and biodegradability, high mechanical strength, less damage to processing equipment, flexibility in processing, high surface finish, low-level health hazards and light weight are preferable properties of natural fibers over synthetic fibers. Flax and hemp fibers are utilized in automotives, construction, packaging and furniture as raw materials. Mercedes applied jute-fiber composites for the door panels of their E-class and banana fiber composites for their A-class vehicles [41]. In 2003, Araco Corporation in Japan created a fully electric vehicle, “Grasshopper”, mainly based on kenaf fibers [42]. The use of kenaf fibers in the door panels of the Ford Mondeo in the UK reduced its weight by 5–10%. However, large variations in quality, depending on the growth rate and absorption of moisture, are considered the biggest disadvantages of natural fiber-based materials. The complementary properties of natural and synthetic fibers, the former being biodegradable and the latter being mechanically strong, have facilitated hybrid composites for numerous applications in recent years. These include sustainable green buildings [43], sustainable agriculture [44], and sustainable automotive and military [45] applications.

- *Health*

Dietary fibers and proteins in seaweeds have nutraceutical and pharmaceutical applications that are beneficial for society and industry [46]. This will help to achieve ecological sustainability, reduce chemical components and control pollution. Polymer-based nanofibers are notable materials in drug delivery, pharmaceuticals, electronics, smart textiles, tissue engineering, etc., which are some of the key applications that will be focused on in Society 5.0 [27]. The type of polymer and its interaction with the drug classify the drug release profiles of nanofibers as immediate, delayed or modified. In a typical drug release, a burst release is accompanied by a linear, sustained release profile. In a core–shell fiber, the core can act as a drug reservoir, and the shell protects and controls the release rate. Antibiotics, anticancer agents, analgesics, and anti-inflammatory drugs are incorporated into nanofibers for oral, oromucosal, transdermal and ocular administration. In the tissue engineering field, nanofibrous scaffolds oriented in specific directions stimulate the transmission and transduction of biochemical and mechanical extracellular signals to the nucleus to enhance specific gene expression, nucleation and growth. Biocompatibility and porous structures of the nanofibers are required criteria for tissue engineering applications. Chitosan, polyvinyl alcohol (PVA), and polyurethane nanofibers loaded with various nanoparticles of silver, emodin, gelatin, etc., were applied for diabetic wound healing.

- *Energy*

Electrospun nanostructures of highly crystalline and hierarchical TiO_2_ of various morphologies (regular fibers, porous rods, hollow tubes and spindles) were applied as photoanodes in dye-sensitized solar cells [47]. Their morphology can vary depending on the annealing temperature (400–800 degrees), and crystallographic phase transformation occurred for TiO_2_. Such materials with high energy efficiency are good alternative materials for energy-related applications. Another appealing application of Fe_2_O_3_ hollow fibers in magnetic materials is electrospinning polyvinylpyrrolidone (PVP)/Fe(NO_3_)_3_ composite solution, followed by calcination [48]. However, smart fibers and textiles provide the best wearable human-mechanical-energy-harvesting systems, overcoming the practical difficulties associated with low-output piezoelectric generators and hard-to-wear electromagnetic devices [49]. Scientists and technologists are developing more knitting machines and professional garments to reduce the damage due to the different elastic moduli of core-spun fiber yarns. Such smart clothing and fibers can well connect AI, machine learning and smart home concepts, further contributing to shaping Society 5.0. Energy management is also supported by introducing battery management systems [50], human interactive sensors and self-powering devices [51].

- *3D Printing*

Sustainability in manufacturing, novel design, a wide scope of materials, cost efficiency and waste remediation are eminent features of 3D printing or additive manufacturing, which is a highly significant method of manufacturing for developing a smart society [52]. The applications of 3D printed materials include printed shoes and clothing, which require flexibility, strength, resilience and ductility. Moreover, 3D printed textile applications offer the freedom to customize the textile shape according to the individual's body shape and size, even by personal scanning. Textiles and garments produced by 3D printing ensure sustainable design, with minimal waste production and reduced energy and transportation costs. Rapid prototyping and single-phase production of this technology provides mass production in a short time and is therefore the best choice for textile manufacturers. The 3D printing of textiles also offers a wide variety of designs according to the designer's choice and enhances the product quality. Thermally conductive knit and woven fibers were developed by 3D printing polyvinyl alcohol and boron nitride nanosheets, thereby developing thermally regulating smart textiles [53]. In fact, 3D printing allows the development of different kinds of fibers, such as yarns, chainmail fabrics, and amimono fabrics. Although some of them have flexibility issues, they are used in robotics, textiles, outer wears, etc. Generally, 3D printing is facilitated in three steps: (1) stereolithography for powder bed fusion; (2) selective laser sintering (SLS), inkjet printing, binder jetting and polyjet for binding; and (3) fused deposition modeling (FDM) and microfiber extrusion for deposition. Smart clothing can also be paired with sensors to monitor heart rate, muscle activities, calorie expenditure, etc.

All the abovementioned fiber technologies will enhance Society 5.0, as they can be combined with body scanning and AI to widen the scope of applications. In addition to well-known challenges in classical fiber technology, such as limitations in material choice and the rigid and stiff nature of applications (e.g., clothing), which can cause discomfort, high-end luxury fashion brands prefer hand crafting compared to 3D printing, which has a high chance of design replication and thus intellectual property infringement. Another classical challenge is the maintenance cost and complex shape formation, which can affect scalable production.

The increased use of fiber technology in Society 5.0 will result in other new challenges to overcome, such as the need to have high data quality for training the AI and preparing the fibers for certain applications. Another challenge is the infrastructure of society and industrial needs, such as computing power and software technology.

In addition to the above technical requirements and fiber properties that need to be used, the strategic, economic and organizational requirements that ought to be implemented to have a real impact on society are noteworthy. This includes the building capacity of the scientist, engineers and technicians who will fabricate these fibers. That is why the preparation of educational systems and, specifically higher education institutions are crucial to any development in Society 5.0.

- *Intelligent fibers*

A separate class of fibers, known as stimulus-responsive fibers, can experience reversible or irreversible changes in their physical, chemical, or mechanical properties when exposed to temperature, pH, light, electrical fields, or mechanical forces [54]. Such fibers find beneficial applications in engineering, electronics, textiles, and medicine. Depending on the nature of stimulation, these smart fibers can be classified into many types: temperature responsive, pH-responsive, light-responsive, etc. While the temperature-responsive or thermoresponsive fibers are based on shape memory polymers and change their shape with temperature variations [55], the light-responsive or photoresponsive (photonic) fibers made of liquid crystal elastomers change their structure or properties when exposed to light [56]. Thermoresponsive fibers are used in drug delivery, self-regulating fabrics, and temperature sensors, and the photonic fibers find applications in smart textiles, optical communications, and biomedical devices. Tu’s research group reports calcium alginate fiber having pH indicating properties to monitor the wound healing process. In the different states of wound healing such as inflammation, and ulceration, the pH value of the wound varies due to internal and external stimuli [57]. The fibers were made by modifying the calcium alginate with hydroxypropyl trimethyl ammonium chloride chitosan, alizarin dye and anthocyanin dye. In addition, intelligent fibers also include electrically responsive, magnetically responsive and mechanically responsive fibers, all exhibiting characteristic property variations when exposed to corresponding stimuli. Finally, the potential for smart fibers to revolutionize various industries and advance the creation of a smart society is enormous.

### General correlation with sustainable development goals

**Fig. 6:**
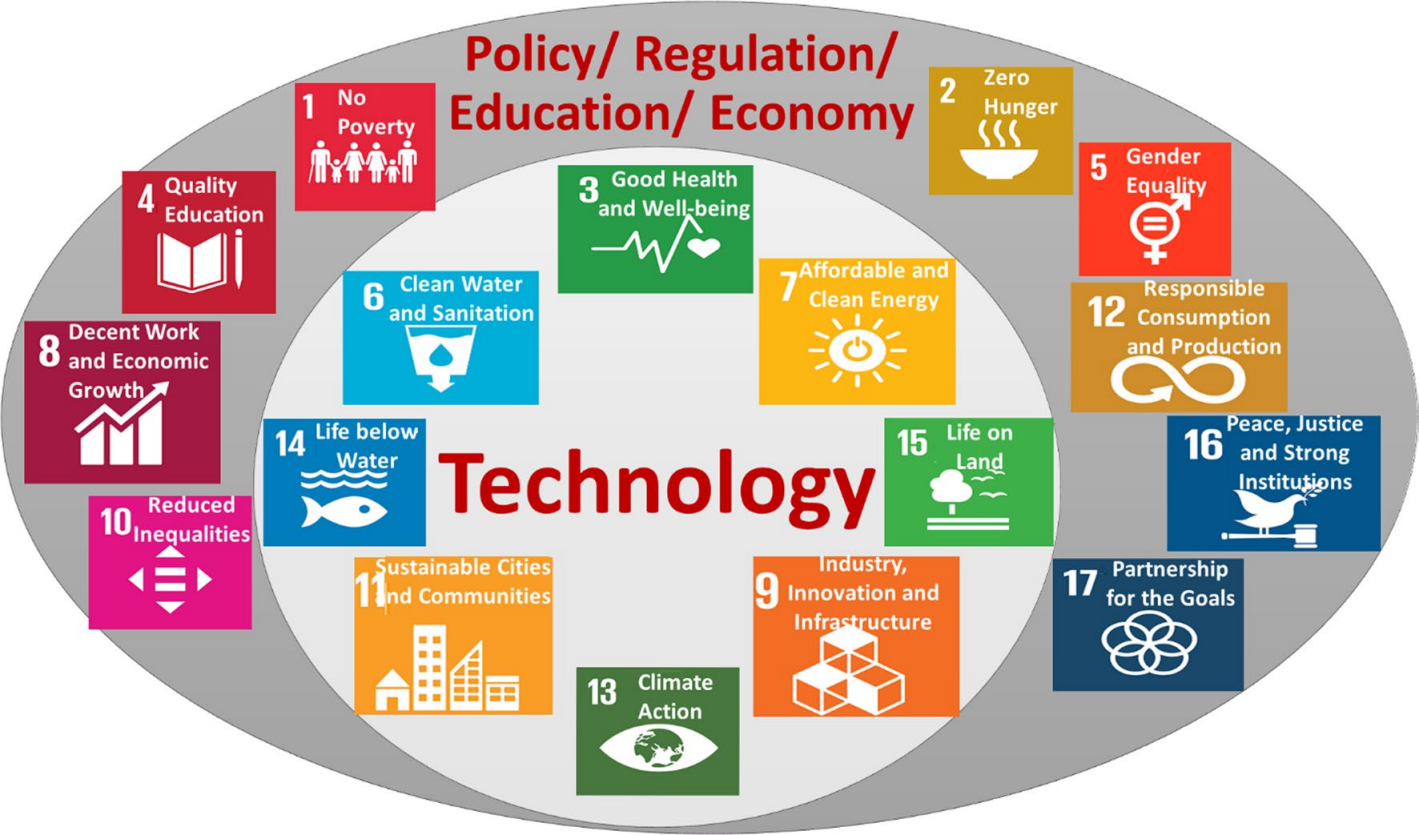
Classification of the direct contributions (inner circle) and indirect contributions (outer circle) of fiber technology to SDGs

The 17 UN sustainable development goals (SDGs) with 169 targets cover sustainability in many fields. In general, fiber technology in industry can be correlated with the SDGs for building sustainably in Society 5.0. Fiber technology has direct and indirect relations with these goals. Figure 6 shows a scheme of the integration of SDGs with fiber technology and the different technological advancements of various fibers.

#### Examples of direct contributions to SDGs

*SDG3: Good health and well-being* Smart fibers that are lightweight and have good deformability are applied in personal health management, including precision therapies, health monitoring, vital biometric checking, and thermal management [58]. This includes graphene fibers responding to different stimuli such as pressure, tension, temperature and humidity, the detection of NO_2_ by Janus graphene/Kevlar textile, and electromyogram monitoring using conductive fibers. Dietary fibers regulate personal health according to the various available sources by interacting with the gut microbiota of the host [59]. Examples of dietary fibers are heterogeneous polysaccharides containing pectin, gum, hemicellulose, cellulose and lignin. Further applicability of fibers in pharmaceutics is discussed in the previous section [46].

*SDG6: Clean water and sanitation for all* To control and overcome water pollution, several agents are utilized to decompose industrial pollutants into harmless wastes. Semiconducting nanomaterials such as TiO_2_, ZnO, α-Fe_2_O_3_, and CuO_2_ have been used to fabricate portable photocatalytic thin films, and one resulting composite was able to achieve maximum degradation efficiency (up to 98%) [60]. Additionally, heavy metals such as Zn^2+^, Hg^+^, Pb^2+^, and Cr^3+^ were removed by chemical precipitation, ion exchange, membrane filtration, and electrochemical technologies using different membranes containing TiO_2_, Al_2_O_3_, bentonite, and carbon nanotubes. Coconut fiber-reinforced polyacrylic acid was used as a superabsorbent hydrogel composite with an equilibrium water absorbency of 342 g/g and maintained superior reswelling ability after several cycles of chemical testing [61]. The hydrogel was applicable in agricultural fields, especially in saline soil, as it was sensitive to salt ions and the ionic strength of the solution. Compared to bare soil, the hydrogel-amended soil had a 29% decreased water requirement.

*SDG7: Affordable and Clean Energy* Hydrogen is a clean energy resource of the future, and the photocatalytic and photoelectrochemical reduction of water produces this fuel. Photoelectrochemical systems composed of cellulose nanofiber-templated TiO_2_ and bacterial cellulose containing Zn_*x*_Cd_1–*x*_S nanoparticles were reported, in addition to a hybrid nanostructure in which ZnO nanorods grew out radially from a cellulose fiber nanogenerator [47]. Graphene fibers with excellent electrical conductivity and easy functionalization were coupled with ferroferric oxide dots through chemical reduction-induced synthesis to achieve an ultrahigh energy density. In ionic liquid electrolytes, nanomaterials favor electrochemical kinetics by changing the valence state of bivalent Fe-trivalent Fe [62]. A wire-shaped supercapacitor fabricated based on this ionogel electrolyte and two linear electrodes produced ultrahigh volumetric energy density, power density and durability over repeated cycles.

**Fig. 7:**
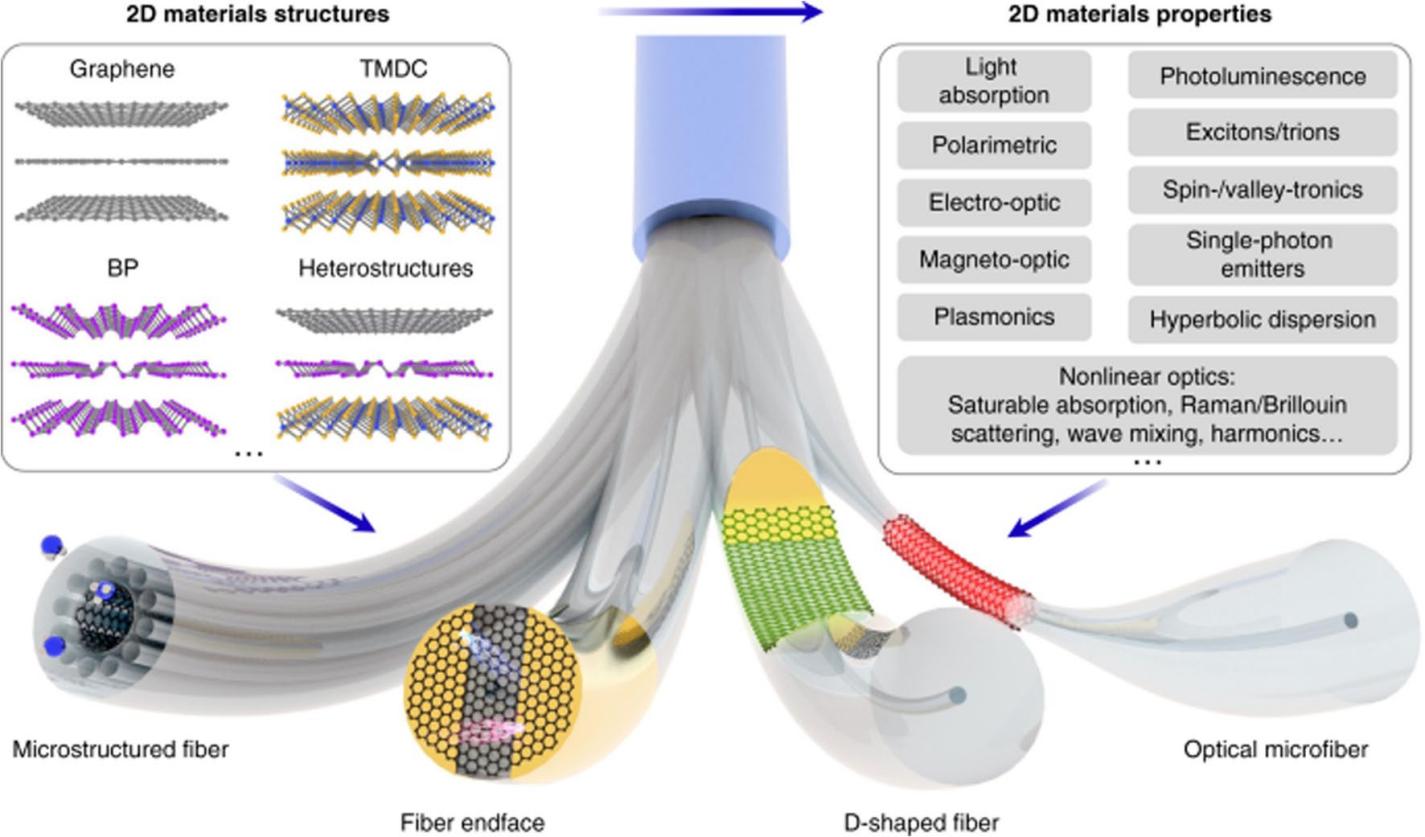
Integration of 2D materials for advanced applications of optical fibers [63]. Reprinted with permission under creative common license

*SDG9: Industry, Innovation and Infrastructure* There are many contributions of fibers in this field; for example, optical fibers are also integrated into 2D materials to enhance fiber photonics and optoelectronics applications [63]. This highly significant area mainly involves fiber optic sensors, graphene polarizers, ultrafast fiber lasers and optical modulator applications. In addition to transition metal dichalcogenides (TMDCs) and black phosphorus, 2D magnets such as CrI_3_ and Fe_3_GeTe_2_ with the magneto-optical Kerr effect have also been applied to develop optical nonreciprocal fiber devices. Carbon fiber surfaces were modified by cardanol functionalization to generate active surface sites to facilitate grafting with unsaturated polyester resins and the resultant composite. Figure 7 summarizes the advanced properties and applications of optical fibers.

*SDG11: Sustainable cities and communities* Civil and architectural designs of a typical sustainable community and society are made to address issues such as flame retardance, carbon emission, energy consumption, reinforcement, cost analysis and experimental verification [64]. Sustainable construction designs are developed by reinforcing plastics/concrete with carbon fibers. In most of these processes, coupling agents with the proper chemical functional groups are used to reduce the interfacial binding forces between the carbon fiber and the matrix used to fill them. Sustainable architectural designs, such as those incorporating coconut husk fibers and recycled tyre steel fibers [65] and foamed hair reinforced clay [66] as is or in concrete medium, provide high heat, sound and moisture insulation capability and improve the resilience of buildings. Moreover, naturally available fibers of plant and animal origin sustain various crafts, which require high-level skills and human efforts. They are the backbones of the economy for different countries, such as Indonesia [67].

*SDG13: Climate Action* Rising levels of CO_2_ in the atmosphere are causing dramatic climatic change, and fiber chemistry and technology are superior with regard to controlling CO_2_. For instance, polymer fibers of hollow or spiral wound geometry, such as cellulose acetate, polysulfone, polyethylene oxide, and silicon rubber, are commercially available for CO_2_ separation. Inorganic zeolites, metal–organic frameworks (MOFs) and carbon were added to polymers to generate polymer matrix composite hollow fibers by reducing the swelling and plasticization effects [68]. Geopolymer-based miscanthus fiber composites [69], recycled PET fiber-reinforced concrete [70], etc., were also identified for CO_2_ neutrality. Mostly, the strength of the bonding between the matrix-fiber interfaces is lower and can be improved by proper modification of the fiber surface.

SDG14: Life below water and SDG15: Life on land are directly related to the various fiber applications mentioned above. All other SDGs have indirect connections with fiber reinforcement and their different composite structures, as demonstrated in Fig. 6.

## Moving ahead

The vision of Society 5.0 is to reframe two different kinds of relationships between society and technology by using digital transformation to achieve a comfortable and high-quality society. It goes beyond industrial revolution to include advanced analytics and people as major elements.

Scientists should understand and contribute to Society 5.0. In this perspective, we show the contributions of fiber technology, which goes beyond health, energy and sustainability, to achieving sustainable development goals and emphasize the need for regulations, policy and education to realize this achievement. Generating and tuning the surface chemistry of fibers is beneficial for resolving most of the current challenges for a sustainable society. However, improving the representations and possibilities of chemical/physical modifications, achieving better functionalities and extending multidisciplinary approaches in fiber-based technology is a long-term goal. It demands greater coordination between the structural information of fibers, cost-effective manufacturing strategies, development of complex and interdisciplinary heterostructures, identification of economic and social well-being, regulation of sustainable environmental conditions, and IoT-based applicability. To move forward, a greater level of integration of fiber technology in applications related to SDGs is needed. Advanced scientific training for students and early career scientists needs to be established. Classical fiber technology production now includes other challenges, such as data training, computer infrastructure and the need for more integration and collaboration between experts in these fields. Scientists, in addition to their focus on their scientific challenges and work in labs, should understand the whole socioeconomic system of Society 5.0 to have a better impact on achieving this society.
